# Surgical-orthodontic retreatment of a severe skeletal Class III malocclusion following an orthodontic camouflage

**DOI:** 10.1590/2177-6709.26.4.e2119247.oar

**Published:** 2021-09-10

**Authors:** Francisco MARTINO, Manuel PEÑA, Rony JOUBERT

**Affiliations:** 1 Universidad del Salvador, Escuela de Postgrados en Odontología, Carrera de Especialización en Ortodoncia (Buenos Aires, Argentina).; 2 Universidad Iberoamericana, Orthodontic Program (Santo Domingo, Dominican Republic).; 3 Universidad Iberoamericana, Oral Surgery Program (Santo Domingo, Dominican Republic).; 4 Universidad Nacional Pedro Henríquez Ureña, Dentistry Program (Santo Domingo, Dominican Republic).

**Keywords:** Orthodontics, Orthognathic surgery, Retreatment, Class III

## Abstract

**Introduction:**

Class III malocclusions are some of the most difficult occlusal anomalies to be treated. Some patients with this condition may require orthognathic surgery, while others may be treated with dental camouflage. Proper patient assessment and selection remains critical in order to achieve favorable results.

**Objectives:**

This report outlines the case of an 18-year-old male who sought retreatment for a severe skeletal Class III dentofacial deformity after undergoing orthodontic camouflage treatment involving mandibular arch extractions. A treatment plan comprising dental decompensation and orthognathic surgery was implemented in order to achieve optimal facial and occlusal results.

**Results:**

After 28 months of treatment, skeletal and dental correction was achieved and facial features were significantly improved. The orthognathic surgery required a 20-mm sagittal maxillomandibular skeletal correction, combined with a 4-mm correction of the midlines and a 2-mm impaction of the maxilla.

**Conclusion:**

Dental compensation may be a risky treatment alternative for severe dentoskeletal discrepancies. In these patients, orthodontics combined with orthognathic surgery is the recommended treatment option.

## RESUMO

**Introdução:**

As más oclusões de Classe III são uma das anomalias oclusais mais difíceis de serem tratadas. Alguns pacientes com essa condição podem precisar de cirurgia ortognática, enquanto outros podem ser tratados por meio da camuflagem ortodôntica. A correta avaliação e seleção do paciente para cada tipo de abordagem permanece uma decisão crítica para se obter resultados favoráveis.

**Objetivos:**

O presente artigo relata o caso de um paciente do sexo masculino, com 18 anos de idade, que procurou tratamento por causa de uma má oclusão esquelética severa de Classe III, após ter se submetido a camuflagem ortodôntica com extração de dentes inferiores. Um plano de tratamento envolvendo descompensação dentária e cirurgia ortognática foi implementado, com a finalidade de atingir resultados faciais e oclusais ideais.

**Resultados:**

Após 28 meses de tratamento, foi alcançada a correção esquelética e dentária, e as características faciais obtiveram uma melhora significativa. A cirurgia ortognática exigiu uma correção esquelética sagital bimaxilar de 20 mm, associada a 4 mm de correção das linhas médias e 2 mm de impacção da maxila.

**Conclusão:**

A compensação dentária pode ser um tratamento alternativo arriscado para discrepâncias dentoesqueléticas severas. Nesses pacientes, a Ortodontia associada à Cirurgia Ortognática é a opção de tratamento recomendada.

## INTRODUCTION

When treating Class III dentofacial deformities in patients with little or no further skeletal growth potential, there are two possible treatment options: orthodontic camouflage or orthodontics combined with surgical repositioning of the jaws.^1^ ^,^ ^2^ ^,^ ^3^ Orthodontic camouflage is viable when treating patients with mild to moderate dentoskeletal discrepancies with acceptable facial aesthetics.^4^ ^-^ ^7^ However, in patients with severe skeletal discrepancies, a combined surgical-orthodontic approach is the preferred method in order to improve facial aesthetics and achieve a stable occlusion.^8^ ^-^ ^10^

Camouflage orthodontic treatment for severe Class III skeletal discrepancies requires excessive compensatory tooth movements to achieve acceptable results, which may end up leading to adverse aesthetic side effects and other problems such as root resorption, periodontal disease and poor stability.^11^ Furthermore, the patient could grow out of the range of successful camouflage treatment, leading to the need for a surgical correction.^12^ If the compensatory treatment plan includes the irreversible step of extracting mandibular premolars, additional space management issues may arise during the pre-surgical orthodontic phase of the retreatment.

In this article, the corrective retreatment of a patient with a severe dental and skeletal Class III is presented. The case previously involved an unsuccessful orthodontic camouflage treatment with extraction of two mandibular premolars. 

## DIAGNOSIS AND ETIOLOGY

**Figure 1:**
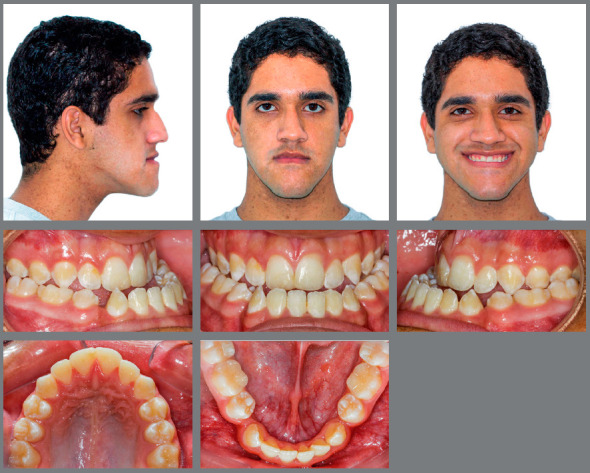
Pre-treatment facial and intraoral photographs. In a failed attempt to compensate the Class III malocclusion, mandibular first premolars were extracted in a previous orthodontic treatment.

An 18-year-old male patient presented for orthodontic retreatment with the chief complaint of unaesthetic facial appearance (Figs 1-4). Previous treatment lasted 24 months and afterwards a retention period of 19 months. During clinical evaluation, a strongly concave profile with accentuated mandibular prognathism and lip incompetence was observed. A severe Class III molar relationship was present, combined with a substantial anterior crossbite (overjet -11 mm) and an excessive retroclination of the mandibular incisors. The mandibular first premolars were extracted during the previous orthodontic treatment, and at this point, 3-mm and 2-mm spaces were present in the right and left extraction sites, respectively. The cephalometric analysis indicated a skeletal Class III pattern due to mandibular prognathism (ANB = -11.5º, SNA = 84.3º, SNB = 95.8º). A substantial retroclination of the mandibular incisors (IMPA = 59º) and a vertical pattern, within the normal parameters (FMA = 24.8º) were also observed (Table 1). Additionally, a transverse asymmetry due to combination of a rotation of the maxilla (2 mm to the right) and the mandible (2 mm to the left) was found.

**Table 1:** Cephalometric data.

Measurement	Norm	Pre-treatment	Pre-surgical	Post-treatment
SNA	82	84.3	84	87
SNB	80	95.8	96.1	85.4
ANB	2	-11.5	-12.1	1.6
FMA	26	24.8	23.6	28.5
IMPA	95	59	90.6	83.5
U1-Palatal Plane	110	114.3	116.6	112.2
Interincisal Angle	130	157.7	126.2	134.2
Lower Lip to E Plane	-2	-0.5	3	-2.1

**Figure 2: f2:**
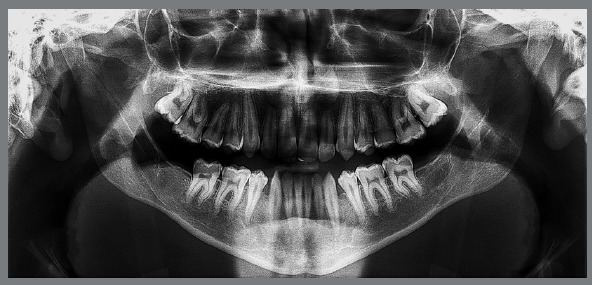
Initial panoramic radiograph.

**Figure 3: f3:**
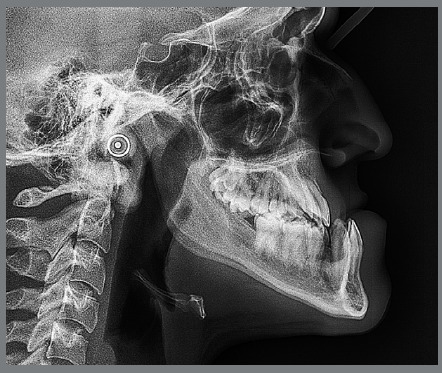
Initial lateral radiograph.

**Figure 4:**
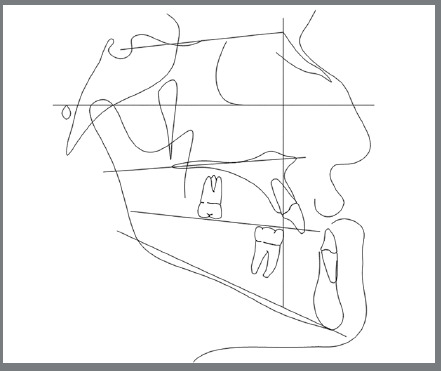
Initial lateral cephalometric tracing.

In the panoramic radiograph, signs of root resorption were observed in the maxillary incisors, and a mild horizontal bone loss was detected in the mandibular incisors. 

### TREATMENT OBJECTIVES

In order to correct the problems identified in this patient, the following objectives were set: buccal movement of the mandibular incisors, to achieve proper uprighting; mandibular setback combined with the advancement and impaction of the maxilla, to improve facial esthetics, achieve dental correction, and enhance incisor display in the smile. Lastly, mandible and maxilla alignment, to correct dental and facial midlines.

## TREATMENT ALTERNATIVES

After reviewing the diagnostic findings, a dental compensatory treatment was discarded, due to the skeletal nature of the Class III deformity. It was concluded that a non-compensatory treatment approach was necessary and consequently, a combined orthodontic and orthognathic surgery treatment plan was proposed, in order to improve facial esthetics and obtain an adequate masticatory function. The pre-surgical orthodontic phase involved the alignment of the dentition within the arches, dental decompensation, leveling of the curve of Spee and coordination of the arches. In order to improve the position of the incisors within the bone bases, it was decided to reopen the mandibular first premolars spaces. The surgical plan included a Le Fort 1 osteotomy for maxillary advancement, impaction and centralization, combined with a bilateral sagittal split osteotomy for mandibular setback and centralization.

## TREATMENT PROGRESS

**Figure 5:**
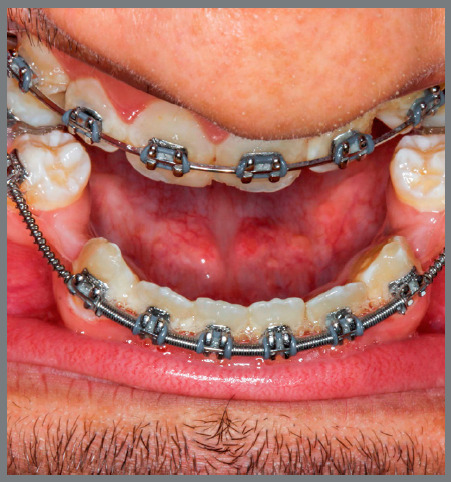
Dental decompensation involved the reopening of the mandibular first premolar spaces. The resulting overjet was -17.0 mm.

Fixed preadjusted appliances were bonded (Roth prescription, 0.022 x 0.028-in slot) and initial leveling and alignment was performed using NiTi round archwires. Subsequently, rectangular stainless steel archwires were placed to coordinate the arches, and the mandibular first premolar spaces were reopened with the use of NiTi coil springs. Decompensation of the mandibular arch occurred by leveling the curve of Spee and the projection of the mandibular incisors, despite lower lip resistance. After 20 months of treatment, the patient was ready for orthognathic surgery. At this point, the resulting overjet was -17 mm (Fig 5).

**Figure 6:**
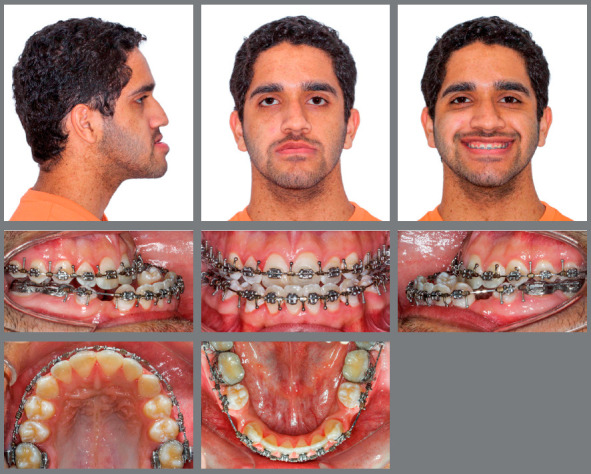
Intermediate facial and intraoral photographs. After 20 months of pre-surgical orthodontic treatment, dental decompensation was achieved.

Pre-surgical records were obtained two weeks prior to surgery, and at the same appointment hooks were placed on passive 0.019 x 0.025-in stainless steel archwires that had been in place for more than six months (Figs 6-8). The orthognathic surgery consisted of 8-mm maxillary advancement with a 2-mm rotation to the left and a 2-mm impaction, combined with 12-mm mandibular setback, with a 2-mm rotation to the right. Due to the magnitude of the mandibular setback, the surgeon chose to use large reconstruction plates for better stability of the bone segments. Post-surgical orthodontic treatment continued for eight months, with the objective of achieving a stable final intercuspation of the teeth.

**Figure 7: f7:**
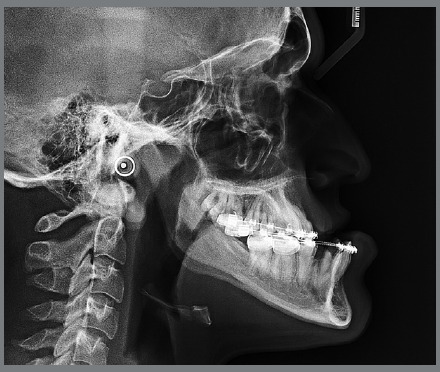
Intermediate lateral radiograph.

**Figure 8:**
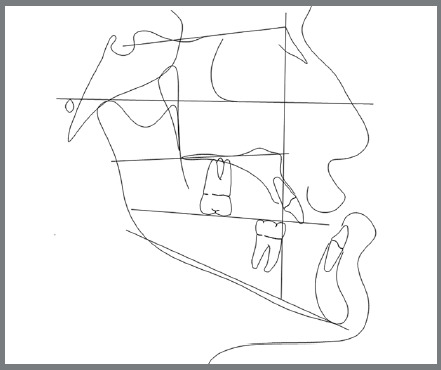
Intermediate lateral cephalometric tracing.

Total treatment time was 28 months and, when combined with the previous camouflage treatment, total time using brackets for this patient was 52 months. For the retention phase, a lower fixed retainer was bonded, combined with an upper Hawley retainer. In order to maintain the space of the mandibular first premolars, temporary fixed retainers were placed and afterwards two fiber-reinforced ceromer-based adhesive bridges were set as temporary space retainers. Dental implants with porcelain crowns were planned as permanent restoration, but the patient decided to postpone this treatment. 

## TREATMENT RESULTS

A 20.0-mm sagittal maxillomandibular skeletal correction was achieved with orthodontic and orthognathic surgery treatment. Facial features dramatically improved, resulting in a straight facial profile, adequate facial symmetry and a harmonious smile. The resulting facial appearance was balanced, aesthetically pleasing and respecting the individual characteristics of the patient. 

**Figure 9:**
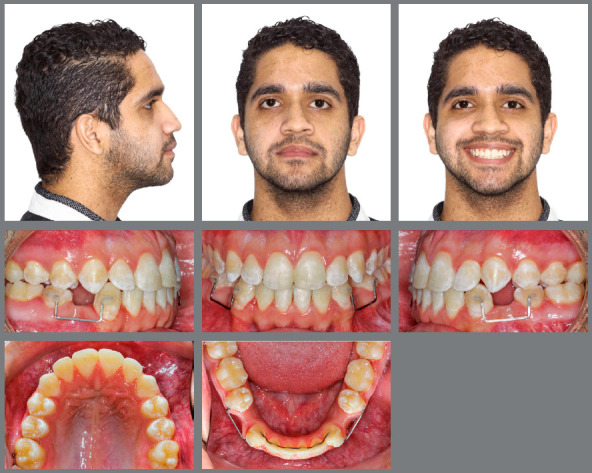
Final facial and intraoral photographs, after 28 months of surgical-orthodontic treatment. The total maxillomandibular sagittal correction was 20.0 mm.

The final occlusion had an acceptable intercuspation and canine guidance. Coincident dental and facial midlines were also attained, and a substantial correction of the overjet was achieved (from -17.0 mm prior to the surgery to 2.0 mm at the end of treatment) (Figs 9-12).

**Figure 10: f10:**
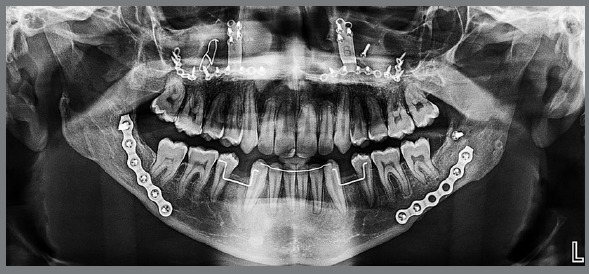
Final panoramic radiograph.

**Figure 11: f11:**
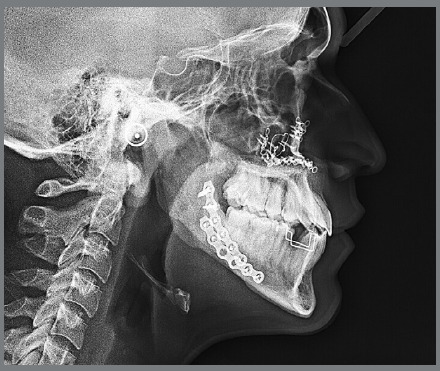
Final lateral radiograph.

**Figure 12:**
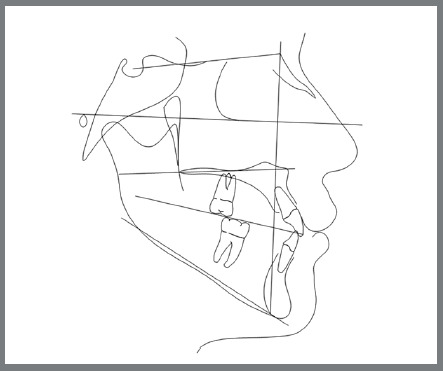
Final lateral cephalometric tracing.

**Figure 13:**
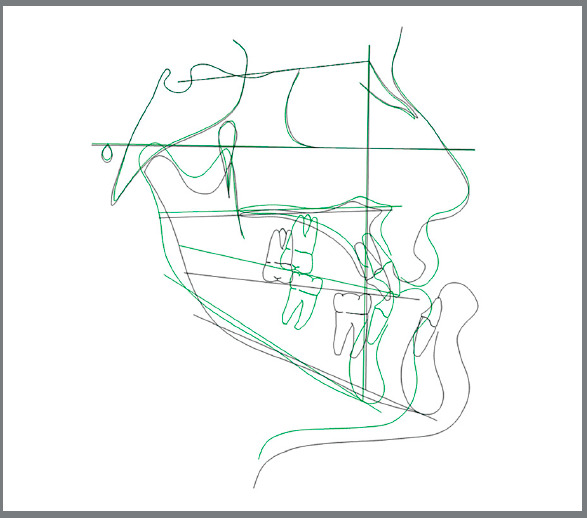
Superimposition of pre-surgical (black) and post-treatment (green) cephalometric tracings.

A comparison between the pre-surgical and final cephalometrics shows that the ANB angle was normalized, increasing from -12.1º to 1.6º. The 8-mm maxillary advancement resulted in a 3º increase of the SNA angle (from 84º to 87º), and the 12-mm mandibular setback induced a 10.7º decrease of the SNB angle (from 96.1º to 85.4º) (Fig 13).

**Figure 14:**
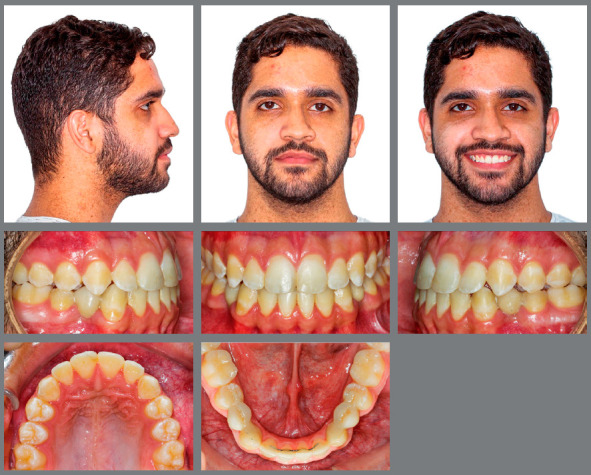
Follow up records one year after debonding. Temporary fiber-reinforced ceromer-based adhesive bridges were bonded to replace the missing lower premolars.

One year after debonding, the results were stable and the patient was pleased with his facial and occlusal outcome (Fig 14).

## DISCUSSION

Camouflage Class III treatment usually relies on the extraction of mandibular premolars to correct the anterior crossbite and disguise the skeletal discrepancy. This treatment approach will typically require an excessive lingual inclination of the mandibular incisors, which can often make the chin appear even more prominent, resulting in an unaesthetic outcome.^13^ Other complications may include root exposure by resorption of the cortical plate, with subsequent gingival recession and fremitus.^14^ ^,^ ^15^ ^,^ ^16^

A study by Kerr et al.^17^ concluded that orthodontic compensation can effectively camouflage the skeletal and dental aspects of the malocclusion if initial ANB is greater than -4.5º and mandibular incisor angulation is greater than 83º. In this case reported, the patient presented for retreatment with an ANB of -11.5º and mandibular incisor angulation of 59º, which combined with poor facial aesthetics, clearly established the need for a surgical-orthodontic retreatment. In order to allow adequate surgical movements, mandibular incisor uprighting was critical; therefore, space reopening in the mandibular arch was necessary during presurgical orthodontics. Options to move mesially the entire mandibular dental arch were considered, but rejected due to the amount of movement required.

Long term stability for this case is a concern, due to the extreme surgical movements that were necessary to correct the skeletal discrepancy in the sagittal plane (8.0-mm maxillary advancement and 12.0-mm mandibular setback). According to a systematic review by Mucedero et al,^18^ bimaxillary surgery for Class III correction will be stable when the maxillary advancement is less than 5 to 6.0 mm, and the mandibular setback, less than 7.0 mm.

Looking at this case retrospectively, the camouflage treatment negatively affected the profile, made presurgical orthodontics more complex, and created the need for permanent restorations. Undergoing a retreatment had a negative psychological impact on the patient, due to undesirable results, time consumption and financial burden. A more careful treatment planning based on an accurate growth analysis and realistic goals may have provided enough information to delay the treatment until the patient had stopped growing and surgery could have been performed.

The study of treatment difficulties, such as those presented in this case report, provides a rare opportunity to gain perspective and aim towards an improvement in the quality of care we provide to our patients.^19^ Inexperience or lack of training are commonly attributed as causes for complications and unfavorable outcomes. Nevertheless, even orthodontists with vast experience and adequate training may also expose their patients to some degree of unintended irreversible damage. In such cases, limited time for diagnosis and treatment planning due to overcrowded offices may play a part.^20^

## CONCLUSIONS

Orthodontics combined with orthognathic surgery is the recommended treatment option for achieving a stable occlusion and facial esthetics in non-growing patients with severe Class III dentoskeletal discrepancies. When considering camouflage treatment for growing patients with this condition, a careful diagnosis is essential in order to develop a customized goal-oriented treatment plan that considers tooth movement limitations, facial characteristics and remaining growth. The presented case highlights the adverse effects that compensatory treatment may have in growing skeletal Class III patients, resulting in the need for a second treatment, combining orthodontics and orthognathic surgery, to achieve optimal treatment results.

## References

[1] Proffit WR, White RP, Proffit WR, White RP, Sarver DM (2003). Contemporary treatment of dentofacial deformity..

[2] Baik HS (2007). Limitations in orthopedic and camouflage treatment for Class III malocclusion. Semin Orthod.

[3] Keim RG (2017). Editor's corner camouflage or surgery?. J Clin. Orthod.

[4] Anhoury P (2013). Retromolar miniscrew implants for Class III camouflage treatment. J Clin Orthod.

[5] Bilodeau JE (2000). Class III nonsurgical treatment a case report. Am J Orthod Dentofacial Orthop.

[6] Popp TW, Gooris CG, Schur JA (1993). Nonsurgical treatment for a Class III dental relationship A case report. Am J Orthod Dentofacial Orthop.

[7] Costa Pinho TM, Ustrell Torrent JM, Correia Pinto JG (2004). Orthodontic camouflage in the case of a skeletal class III malocclusion. World J Orthod.

[8] Stellzig-Eisenhauer A, Lux CJ, Schuster G (2002). Treatment decision in adult patients with class iii malocclusion orthodontic therapy or orthognatic surgery?. Am J Orthod Dentofacial. Orthop.

[9] Troy BA, Shanker S, Fields HW, Vig K, Johnston W (2009). Comparison of incisor inclination in patients with Class III malocclusion treated with orthognatic surgery or orthodontic camouflage. Am J Orthod Dentofacial Orthop.

[10] Rino J, de Paiva JB, Miasiro H, Attizzani MF (2007). Surgical-orthodontic treatment of a class iii dentofacial deformity. J Clin Orthod.

[11] Liu X, Yang Z (2010). Orthodontic camouflage treatment of an adult skeletal class III malocclusion. J Clin Orthod.

[12] Burns NR, Musich DR, Martin C, Razmus T, Gunel E, Ngan P (2010). Class III camouflage treatment: what are the limits. Am J Orthod Dentofacial Orthop.

[13] Proffit WR, Sarver DM, Proffit WR, White RP, Sarver DM (2003). Contemporary treatment of dentofacial deformity..

[14] Kaley J, Phillips C (1991). Factors related to root resorption in edgewise practice. Angle Orthod.

[15] Sperry TP, Speidel TM, Isaacson RJ, Worms FW (1977). The role of dental compensations in the orthodontic treatment of mandibular prognathism. Angle Orthod.

[16] Hisano M, Chung CR, Soma K (2007). Nonsurgical correction of skeletal Class III malocclusion with lateral shift in an adult. Am J Orthod Dentofacial Orthop.

[17] Kerr WJ, Miller S, Dawber JE (1992). Class III malocclusion surgery or orthodontics?. Br J Orthod.

[18] Mucedero M, Coviello A, Baccetti T, Franchi L, Cozza P (2008). Stability factors after double-jaw surgery in Class III malocclusion A systematic review. Angle Orthod.

[19] Beherents RG (1996). Iatrogenics in orthodontics. Am J Orthod Dentofacial Orthop.

[20] Barreto GM, Feitosa HO (2016). Iatrogenics in orthodontics and its challenges. Dental Press J Orthod.

